# The burden of oral conditions among adolescents living with HIV at a clinic in Johannesburg, South Africa

**DOI:** 10.1371/journal.pone.0222568

**Published:** 2019-10-16

**Authors:** Yolanda Malele Kolisa, Veerasamy Yengopal, Khumbo Shumba, Jude Igumbor

**Affiliations:** 1 School of Oral Health Sciences, Department of Community Dentistry, University of the Witwatersrand, Johannesburg, South Africa; 2 School of Public Health, University of the Witwatersrand, Johannesburg, South Africa; University of Bern, SWITZERLAND

## Abstract

**Background:**

There are inconsistent reports on the prevalence of oral conditions and their associated factors among adolescents living with HIV (ALHIV). The current inconsistencies may hinder the development of clear guidelines on the prevention and treatment of oral conditions among ALHIV. This study provides an update on oral conditions and their associated factors in a cohort of South African ALHIV and receiving routine HIV treatment services at a Johannesburg HIV wellness clinic.

**Methods:**

Decayed Teeth (DT), Decayed Missing and Filled Teeth (DMFT) and Oral HIV/AIDS Research Alliance case definitions were used for caries examination and reporting of the Oral Mucosal Lesions (OML) respectively. Data analyses were stratified by the study main outcomes; chi-squared tests were performed to determine the associations; and multiple logistic regressions were also used to identify associated factors after adjusting for other exposure variables. In addition to fitting logistic regressions, we explored the data for potential confounders and effect modifiers.

**Results:**

A total of 407 ALHIV were assessed, of which 51.0% were females. The mean age of the ALHIV was 14.75 years (SD 2.43) while the median age of their parents was 43 years (IQR 37–48 years). Regardless of sex, age group and other socio-demographic characteristics, participants had high count of dental caries (DMFT>0). The overall prevalence of dental caries was 56.76% (n = 231) with mean DT score of 2.0 (SD 2.48) and mean DMFT score of 2.65 (SD 3.01). Dental caries prevalence (DT>0) was significantly associated with the HIV clinical markers. HIV RNA viral loads more than 1000 copies/ml and CD4 cell counts less than 200 count cells/mm^3^, increased the likelihood of having dental decay among ALHIV (p<0.05). ALHIV at WHO staging III, IV had higher caries prevalence ranging from 70% to 75% (p<0.05). The prevalence of dental caries was directly related to the presence of oral mucosal lesions (p<0.05). The prevalence of OML was 22%, with linear gingival erythema (13.8%) accounting for most of the OML. Multiple logistic regression modelling suggested that dental caries experience (DMFT>0), age category 13–15 years, WHO staging of IV and viral load of more than 1000 copies/ml significantly predicted the outcome of oral lesions as assessed using the OHARA case definitions (p<0.05). The odds of developing dental caries was also 1.5 times more among ALHIV who brush their teeth less frequently and those who reported more frequent eating of sugar sweetened diets (p<0.05).

**Conclusions:**

There is high prevalence of dental caries and OML among ALHIV in Johannesburg. The reported prevalence was associated with high HIV RNA viral loads, shorter duration on antiretroviral treatment and high WHO staging of HIV disease on crude analysis. Additionally, caries experience contributed to the prevalence of OML. Our study acknowledges the protective effect of HIV treatment and positive oral health practices on the presence of oral conditions among ALHIV in Johannesburg.

## Introduction

The chronicity of HIV infection has been associated with varying patterns and presentations of oral diseases mainly linked to whether the patients were on antiretroviral treatment (ART) or not. For example, the long-term use of ART has significantly decreased the presence of opportunistic lesions commonly associated with HIV infection [1, 2]. The prevalence of oral conditions has also been attributed to other treatment characteristics such as the regimen [2]. In the era before the widespread availability and use of ART, oral lesions were considered as one of the first signs of HIV seropositivity, and an indication of treatment failure and/or poor adherence to ART [3–5]. Furthermore, the occurrence and type of lesions before and during ART have also been reported to differ in different settings [2].

Despite the improved and increasing use of ART, oral lesions associated with HIV infection have been reported to continually persist across all age groups among children [5–7]. For instance, research studies in India and Uganda have reported over 60% prevalence of orofacial manifestations associated with HIV among children and adolescents who were on ART[7, 8]. Common lesion identified among ALHIV include ulcers, warts, carcinoma and salivary gland conditions [2]. This trend is a source of concern for South Africa, because the country maintains the highest number of people living with HIV (PLHIV) globally, including its approximately 370 000 adolescents aged between 10 and 19 years old, living with the virus [9]. This sub-population is key in this paper and, will be referred to as adolescents living with HIV (ALHIV).

Managing ALHIV on ART presents extra challenges which may influence the occurrence of oral lesions. For example, the disclosure of HIV status to perinatal HIV-infected adolescents may present additional mental health implication and effects. This is given the mean age of disclosure in South Africa to be at about 10 years of age [10]. In addition, treatment fatigue [11] and depression [12] are some of the challenges experienced ALHIV. These factors are known to influence adherence to treatment [13]. Further research suggests that poor treatment adherence among ALHIV, increases the risk of developing caries, periodontal disease and halitosis [4, 14]. Other studies have shown that the use of sugary medicine (syrups) and sugar sweetened diets presents additional risks to oral conditions [15, 16].Other risk factors for oral conditions among adolescents includes poor oral hygiene, social and demographic barriers, periodontal diseases and irregular access to dental services [6, 7, 17].

An epidemiological update of oral conditions among ALHIV, especially in contexts with elevated HIV prevalence, such as South Africa is imperative given the variations in risks factors and prevalence of oral conditions among ALHIV. The goal of this study was to determine the burden of oral disease and their associated factors among ALHIV who are on ART treatment and attending routine HIV treatment and care at a public HIV wellness clinic in Johannesburg, South Africa. In this regard, the presence of oral condition was measured using the Decayed, Missing and Filled Teeth (DMFT) index [18] and the Oral HIV/AIDS Research Alliance (OHARA) case definitions were used to record the oral mucosal conditions [19].

## Methods

### Design and study participants

We conducted a cross-sectional study to determine the burden of oral conditions among ALHIV. The study was conducted among ALHIV aged 10–19 years, on ART and accessing routine HIV treatment, care and support services at a HIV wellness center in Johannesburg, South Africa. The catchment population is made up of inhabitants of the Greater Johannesburg Metropolitan Area. Twenty-one percent of inhabitants in this province are living with HIV [20].

### Sampling

A sample size of 400 participants was calculated. The sample size was based on the assumption that if approximately a quarter (25%) of the participants will have a controlled viral load /virological control (viral load less than 40 copies/ml), then this sample size will have 87% power to detect as statistically significant an absolute difference of 15% in the proportion of participants with oral lesions. The above assumes that amongst the participants with virological control, 15% have oral lesions. So, amongst participants without the viral load control, 30% have oral lesions. The inclusion criteria was: Those who were 14–19 years and voluntarily provided written assent and consent to the study. Parental consent for those aged 14–17 years of age. Participants should be part of the HIV wellness center with confirmed HIV diagnosis and have been initiated on ART. Adolescents aged between 18–19 years did not require parental consent because they were eligible to provide their own written consent. Eventually 415 participants attending the HIV Wellness Clinic were recruited.

### Data collection

Study participants were recruited from the HIV wellness clinic. The participants were approached about the oral health study by the administrative staff at the clinic. During the oral health care routine consultations, potential participants and their caregivers were informed about the details of the study. Those who provided informed consent or/and assent were enrolled as research participants. Recruitment process took approximately nine months, until the estimated sample size was achieved. Eight out of 415 did not agree to participate in the study due to other commitments (not having time for questionnaire and examination due to personal commitments for participants or the parent/caregiver). Information on their socio-demographic characteristics and detailed oral health history were collected. Details of oral health related complaints, oral hygiene habits, and general oral health were collected from participants. Dental examinations were performed by two calibrated dental practitioners. Reports on clinical characteristics such CD4 count, viral load and WHO HIV clinical staging were obtained from participants’ medical records. These variables constitute the clinical markers of HIV disease in our study.

A dental clinical examination based on Decayed Missing and Filled Teeth (DMFT) index as outlined in the primary survey methods by the World Health Organization was also performed[18]. The DMFT index determined the presence of dental caries and treatment needs. Patients were examined while sitting in the supine position on a portable dental chair using artificial light in the clinic. The Oral HIV/AIDS Research Alliance (OHARA) case definitions were used to record the oral mucosal conditions [19]. A trained clinician using the training material slides by based on the 2009 Oral HIV/AIDS Research Alliance (OHARA) recommendation [19]; the score for correctly diagnosed conditions had to be 80% and above in order to proceed with data collection. The inter-examiner reliability was done by re-examining one-tenth of the sample by each examiner, and the calculated kappa statistics was 0.81 for DMFT, and 0.87 for OHARA case definitions.

All statistical analyses were performed using Stata version 14 (StataCorp, College Station, Tx). In the descriptive analysis socio-demographic, oral and clinical characteristics -which were all categorical variables, were summarised using proportions. All analyses were stratified based on the outcome variables of interest: DMFT (caries experience), DT (dental caries) and OHARA (Oral mucosal lesions). Chi-squared tests were performed for associations. We also calculated the Significant Caries Index (Sic) which brings to focus the individuals with the highest caries scores (D) in each population under study. The Sic Index is calculated by selecting one third of the population with the highest DMFT scores. The mean DMFT for this subgroup is calculated. According to the WHO, this Sic Index value provides information on individuals at highest risk and this value should be targeted to be 3 or less [21]. Multiple logistic regression analyses were also used to identify associated factors after adjusting for other exposure variables. Using backward and forward stepwise elimination exposure, variables that had a p-value <0.1 in there crude bivariate regression analyses were included in the multivariate logistic regression modeling were used. In addition to fitting logistic regressions, we also explored the data for potential confounders and effect modifiers. For inferences the significance level was set at 5%. This study received ethical clearance [Ref. number: M161142] from the Human Research Ethics Committee of the University of Witwatersrand, Johannesburg.

## Results

### Socio-demographic characteristics and caries experienced

A total of 407 ALHIV were assessed, of these 51.0% ALHIV were females and 49% males. The participants’ median age was 15 years (IQR 13–15) whilst the age of the parents/caregivers was 43 years old (IQR 37–48). Most of the parents/caregivers were females (78.3%), employed (60.3%), with a secondary school level of education (67.3%). Most of the participants came from families whose household size was composed of four to six people (52.9%). As shown in Table 1; regardless of sex, age group and other socio-demographic characteristics, adolescents had high counts of dental caries (DMFT>0).

**Table 1:** Socio-demographic characteristics of participants with or without caries experience.

Characteristics		Caries experience (DMFT)	
	Sample size(n)^*†*^	DMFT = 0;—n (%)	DMFT>0; n (%)
**Adolescents’ sex**			
Male	196	74(38.14)	120(61.86)
Female	204	75(37.63)	127(62.87)
**Adolescents’ Age Group**			
10-12years	84	38(45.24)	46(54.70)
13–18 years	158	45(28.48)	113(71.52)
16–18 years	154	64(41.56)	90(58.44)
**Adolescents’ Education**			
Primary	137	51(37.23)	86(62.77)
Secondary /Tertiary	237	90(37.97)	147(62.03)
Special School	4	0(0)	4(100)
**Parents’/caregivers’ Sex**			
Male	83	29(35.37)	53(64.63)
Female	300	115(38.72)	182(61.28)
**Parents’/caregivers’ Age group**			
18-30yrs	23	5(22.73)	17(77.27)
31-40yrs	116	39(33.62)	77(66.38)
41-50yrs	154	63(41.18)	90(58.82)
51-60yrs	44	21(48.84)	22(51.16)
> 60yrs	21	4(19.05)	17(80.95)
**Parents’/caregivers’ Employment status**			
Employed	211	65(31.40)	142(68.60)
Unemployed	131	56(42.75)	75(57.25)
Retired	8	4(50)	4(50)
**Parents’/caregivers’ Education**			
Tertiary	77	34(44.16)	43(55.84)
Secondary school	239	86(36.29)	151(63.71)
Primary school	39	15(38.46)	24(61.54)
**Household size**			
1–3 persons	125	40(32.52)	83(67.48)
4–6 people	201	77(38.50)	123(61.50)
>7 People	54	24(45.28)	29(54.72)

^†^ Missing values not included in the totals

### Dental caries

#### Dental caries prevalence and HIV clinical markers

Out of the 407 ALHIV in our study, the caries prevalence was 56.76% (n = 231) with mean ‘D’ (decayed teeth) score of 2.0 (±SD 2.48) and caries experience mean DMFT score of 2.65(±SD 3.01). This means the dental caries component (‘D-decayed’ 2.0) was the major contributor (77%) to the total DMFT score (2.6). The Sic value for the participants was found to be 4.90 (SD = 2.10) which implies that the distribution of dental caries in this study cohort was unevenly skewed towards a third of the individuals in the sample.

Table 2 also presents prevalence and association between dental caries and the clinical markers of HIV disease (including CD4 counts, viral load and the WHO clinical staging of the disease). ALHIV with oral mucosal lesions had higher dental caries compared to those without OML (73% vs 52%, p <0.001). All HIV clinical markers were associated with dental caries. The prevalence of dental caries was higher among those with lower CD4 cell counts (p <0.001). Higher HIV RNA viral loads levels had higher prevalence of dental caries compared to lower HIV RNA viral loads levels (p <0.05). Similarly, the higher the WHO staging (Stages III, IV), the higher the caries prevalence (ranging from 70% to 75% (p<0.05). There was an inverse relationship between dental caries and duration on ART. A decrease in the dental caries prevalence was associated with the increase in the duration on ART from 75% to 44% in decrease (p<0.001) (Table 2).

**Table 2:** HIV clinical markers in ALHIV with and without dental caries.

Characteristic	Dental Caries prevalence		†p-value
	DT = 0 (n = 176) Frequency (%) (percentage)	DT>0 (n = 231) Frequency (%)	
**Caries Experience**			
DMFT = 0	150(100)	0	<0.001***
DMFT>0	26(10.12)	231(89.88)	
**CD4 count**			
<200CD4 cells/mm^3^	13(30.95)	29(69.05)	0.01**
200-500CD4 cells/mm^3^	30(33.33)	60(66.67)	
>500CD4 cells/mm^3^	127(48.29)	136(51.61)	
**Viral Load**			
<40VL copies/ml	62(48.00)	67(51.94)	0.02*
40-1000VL copies/ml	99(44.00)	126(56.00)	
>1000VL copies/ml	12(25.00)	36(75.00)	
**YEARS ON ART**			
0–4 years	18(25.00)	54(75.00)	<0.001***
5–9 years	60(37.50)	100(62.50)	
≥ 10 years	98(56.00)	77(44.00)	
**Oral mucosal lesions**			
OHARA = 0	152(47.65)	167(52.35)	<0.001***
OHARA >0ᵃ	24(27.27)	64(72.73)	
**WHO HIV STAGING**			
I	113(52.80)	101(47.20)	<0.001***
II	15(39.47)	23(60.53)	
III	19(29.69)	45(70.31)	
IV	19(25.33)	56(74.67)	

OHARA>0

ᵃ referred to at least one lesion diagnosed.

† Based on Pearson chi-squared tests

* *p* ≤ 0.05

** *p* < 0.01

*** *p* < 0.001

Unadjusted logistic regression shown in Table 3 indicated that HIV RNA viral loads greater than 1000 copies/ml, WHO stage III and IV, increased the odds of dental caries by more than 2.5 times, than viral undetectable loads and WHO Stage I and II respectively. In contrast, CD4 count cells/mm^3^ of more than 500, a longer duration on ART of more than 10 years reduced the odds of dental decay (OR:0.480[0.239,0.964] and 0.262[OR: 0.142,0.483])(Table 3). However, after adjusting for confounders, only the HIV clinical marker of HIV RNA viral loads more than 1000 copies/ml influenced the odds of dental caries in Table 3. Sex of adolescent, parents’ age group or level of education were not associated with presence of oral conditions.

**Table 3:** Factor associated with dental caries and oral mucosal lesions amongst adolescents living with HIV.

	Dental caries DT>0		OHARA >0*^a^*	
	Unadjusted OR (CI)	Adjusted OR (CI)	Unadjusted OR (CI)	Adjusted OR(CI)
**Viral Load copies/ml**				
<40	Reference	Reference	Reference	Reference
40–1000	1.21[0.79,1.88]	1.24[0.58,2.65]	1.42[0.81,2.49]	1.42[0.80,2.55]
>1000	2.54^**^[1.28,5.06]	1.07[0.26,4.43]	2.37^*^[1.15,4.92]	2.94^*^[1.25,6.92]
**CD 4 counts cell/m**^**3**^				
<200CD4	Reference	Reference	Reference	Reference
200-500CD4	0.90[0.41,1.97]	1.39[0.34,5.77]	1.01[0.43,2.37]	1.11[0.44,2.83]
>500CD4	0.48^*^[0.24,0.96]	0.52[0.15,1.78]	0.82[0.38,1.78]	1.06[0.44,2.56]
**WHO staging**				
I	Reference	Reference	Reference	Reference
II	1.72[0.85,3.47]	1.30[0.36,4.65]	1.56[0.74,3.33]	1.34[0.58,3.01]
III	2.650^**^[1.46,4.83]	1.79[0.70,4.60]	0.95[0.48,1.86]	1.03[0.51,2.09]
IV	3.30^***^[1.84,5.92]	3.93^**^[1.47,10.50]	0.62[0.31,1.23]	0.55[0.26,1.17]
**ALHIV age group**				
10–12 yrs	Reference	Reference	Reference	Reference
13–15 yrs	1.69[0.99,2.89]	0.63[0.22,1.81]	1.16[0.60,2.26]	0.83[0.41,1.66]
16–18 yrs	1.06[0.62,1.81]	0.29^*^[0.09,0.88]	1.35[0.70,2.61]	1.02[0.49,2.11]
**ALHIV Education level**				
Primary	Reference	Reference	Reference	
Secondary/Tertiary	0.87[0.56,1.33]		1.34[0.79,2.29]	
Special School			14.12*[1.408,141.7]	
**Years on ART**				
0–4 yrs	Reference		Reference	
5–9 yrs	0.56[0.30,1.04]		2.59*[1.19,5.65]	
> = 10 yrs	0.26^***^[0.14,0.48)		1.94[0.88,4.25]	
**Caries Experience**				
DMFT = 0			Reference	
DMFT>0			3.03^***^[1.71,5.38]	
**Parents Sex**				
Male	Reference	Reference	Reference	
Female	0.90[0.55,1.478]	0.41*[0.18,0.92]	0.98[0.54,1.77]	
**Parents Employment Status**				
Unemployed	Reference	Reference	Reference	
Employed	1.60^*^[1.03,2.50]	2.85^**^[1.33,6.19]	1.54[0.88,2.72]	
Retired	0.99[0.24,4.11]		5.24*[1.21,22.61]	
**Satisfaction with Oral Health**				
Disagree	Reference	Reference	Reference	
Undecided	1.03[0.67,1.59]	1.42[0.63,3.23]	1.03[0.62,1.70]	
Agree	0.37^**^[0.19,0.73]	1.09[0.29,3.95]	0.63[0.26,1.51]	
**Self-rated oral health**				
Poor-fair	Reference	Reference	Reference	
Good	0.46^***^[0.29,0.71]	0.692[0.30,1.60]	1.17[0.72,1.90]	
Very good-Excellent	0.13^***^[0.064,0.26]	0.17^**^[0.05,0.62]	0.20^**^[0.06,0.66]	
**Soft drinks ingestion**				
Weekly	Reference	Reference	Reference	
Daily	1.51^*^[1.01,2.26]	1.71[0.82,3.55]	1.17[0.71,1.91]	
**Frequency of eating sweets**				
Weekly	Reference		Reference	
Daily	1.51^*^[1.01,2.26]		1.17[0.71,1.91]	
**Tooth brushing frequency**				
Twice daily	Reference	Reference	Reference	
Once daily	1.54^*^[1.03,2.31]	1.73[0.82,3.62]	1.09[0.67,1.77]	

OHARA>0*^a^* referred to at least one lesion diagnosed.

Exponentiated coefficients; 95% confidence intervals in brackets

* *p* < 0.05

** *p* < 0.01

*** *p* < 0.001

^‡^ Marginally significant *p* < 0.1

#### Dental caries and behavioural traits

Bivariate analysis between dental caries experience and behavioural characteristics showed significant association particularly with self-perceived dental treatment need (p≤0.05); self-rated oral health (p<0.01); oral hygiene habits (p<0.05) and sugary diets (≤0.05) as presented in Table 4. The results suggest that those with good oral hygiene behavioural habits were associated with less dental caries occurrence.

**Table 4:** The behavioural characteristics and self-rating of adolescents and the association with untreated caries prevalence.

Behavioural characteristics	DT = 0(%)	DT>0*^a^* (%)	†p-value
**General self-rating of teeth and mouth**			
Poor-fair	51(29.31)	123(70.69)	<0.001
Good	82(47.67)	90(52.33)	
Excellent	42(76.36)	13(23.64)	
**Satisfaction with tooth and mouth condition?**			
Disagree	73(40.11)	109(59.89)	0.01*
Undecided	65(39.39)	100(60.61)	
Agree	29(64.44)	16(35.56)	
**Need dental Treatment?**			
Disagree	55(46.61)	63(53.39)	0.05*
Undecided	35(55.66)	28(44.44)	
Agree	86(38.91)	135(61.09)	
**Tooth-brushing Frequency**			
Once a day	94(48.21)	101(51.79)	0.04*
Twice a day	75(37.88)	123(62.12)	
**Soft drinks consumption frequency**			
Sweet drinks = Weekly	84(48.28)	90(51.72)	0.05*
Sweets drinks = Daily	83(38.25)	134(61.75)	
**Sweets consumption Frequency**			
Sweets = Weekly	84(48.28)	90(51.72)	0.05*
Sweets = Daily	83(38.25)	134(61.75)	
**Total**	42.71%	57.29%	

DT>0*^a^* referred to at least one caries lesion diagnosed.

* *p* ≤ 0.05

† Based on Pearson chi-squared tests.

Unadjusted logistic regression in Table 3, indicated that those who self-rated their oral health to be good/very, good/excellent were less likely to have dental decay (OR: 0.46[0.292, 0.709] and OR: 0.13[0.0636, 0.259) respectively compared to those who rated themselves poor-fair. Frequency of tooth-brushing also determined the occurrence of dental caries. The odds of developing dental caries were 1.5 times more amongst those who reported to less frequent tooth brushing than those who brushed twice daily (OR: 1.542 [1.031,2.305]). The odds of having dental caries were 1.5 more for those with daily consumption of sugar sweetened diets than those with weekly consumption. After adjusting for confounders, only self-rating was likely to be associated with dental caries (OR: 0.1680.0459, 0.616).

### Oral mucosal lesions

#### Oral mucosal lesions (OML) and HIV clinical markers

**Fig 1:**
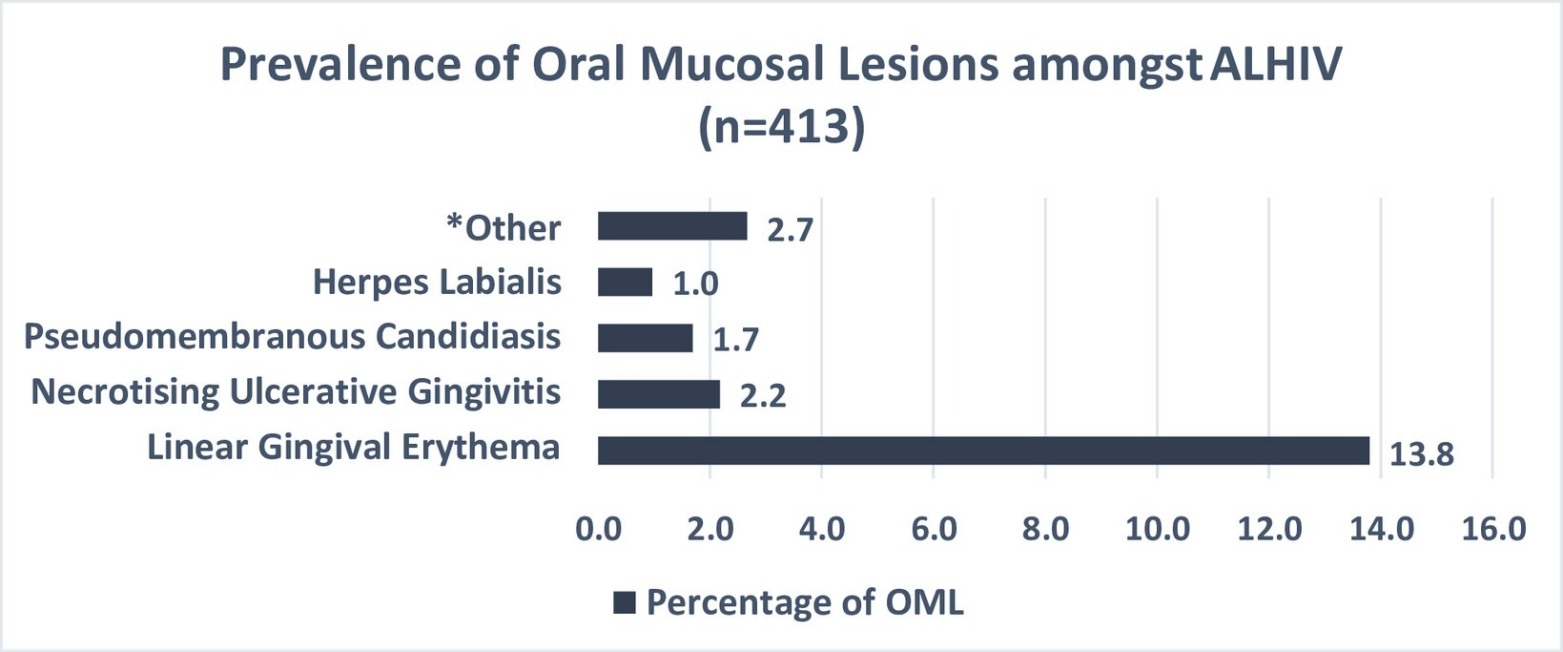
Prevalence and type of oral mucosal lesions using OHARA case definitions.

About 21.2% of the participants had a type of oral mucosal lesions. Fig 1 shows linear gingival erythema (LGE) to be the most commonly diagnosed OML (13.8%), followed by necrotising gingivitis (2.2%) and pseudomembranous candidiasis (1.7%). Other lesions that were diagnosed included herpes labialis n = 4, angular cheilitis n = 3, ulceration not specified and oral warts each n = 2.

#### Oral mucosal conditions and behavioural traits

Results of the unadjusted logistic regression analysis suggested that dental caries experience (DMFT>0), viral load of more than 1000 copies/ml and self-rated oral health that is ‘very good-Excellent’ affected the odds of oral mucosal lesions (OHARA>0) appearance (Table 3). After adjusting for socio-demographic, behavioural and clinical markers confounding; only HIV RNA viral loads of more than 1000 copies/ml increased the odds of oral mucosal lesions occurrence nearly 3 times (OR: 2.935*[1.245,6.920]).

## Discussion

We found 57% prevalence of dental caries among ALHIV on ART. The prevalence of dental caries in our study population is higher than the earlier reported 30% and 42% national dental caries prevalence among 12 and 15 year old respectively in the general population [22]. Our find is however similar to the 61% dental caries prevalence found among a similar American cohort of ALHIV on ART [6]. Furthermore, the untreated mean DT score of 2.2 in the American study is comparable to our study’s DT score of 2.0 where DT contributed 77% to the total DMFT. The latter implies the factors such as attitudes towards dental decay (presence of disease but adolescents wait for symptoms to develop before accessing care) or dental access (adolescents are aware of decayed teeth in the mouth but access to treatment might be a challenge) maybe similar in the two settings. In consonance with our findings, the high prevalence of active dental caries among ALHIV on ART has also been attributed to the sugar sweetened diets and other socio-economic characteristics [6, 17]. Elsewhere, authors have further purported that dental caries maybe related to xerostomia that is induced by the use of ART [4, 14]. Dios and Scully (2014) add that xerostomia is associated with the nucleoside reverse transcriptase inhibitor based ART regimen [4].

The socio-behavioural, clinical, treatment needs, and access related correlates and distribution of dental caries found in our study suggest multi-factorial causes of dental caries and the need for multi-level prevention and treatment interventions. It is evident that the factors that put patients at higher risk to caries in general non-HIV-patients also apply to this HIV positive cohort and should not be ignored. This position is validated by studies that have found socio-economic factors and oral health behavioural characteristics to be strong predictors of the presence of dental caries among adolescents [23, 24]. Our observation of higher prevalence of dental caries among adolescents with younger working parents with the household size of one to three people, may be indicative of the effect of income and social support on the presence of caries among ALHIV. This finding re-emphasizes the need for multi-level prevention and treatment interventions including school oral health programmes, restriction of marketing or vendors selling sugary diets especially within school premises and improvement on access to oral health care for ALHIV.

ALHIV with advanced HIV disease based on WHO clinical staging of HIV disease and high HIV RNA viral loads, were more likely to have dental caries in the current study. In addition, longer HIV treatment duration may have had a protective effect against dental caries. These findings parallel other studies that have found correlations between dental caries and ALHIV immunological profile [25]. Similarly, Beena (2011) found that the dental caries worsened with the deterioration of children’s immunocompetence evidenced by high HIV RNA viral loads.

We also found caries prevalence to be inversely related to regular dental access, good oral health self-rating, and regular brushing of teeth. In our analyses, infrequent brushing of teeth seems to increase the likelihood of having dental caries. Our results therefore support and add to the current evidence that good oral hygienic habits and regular access to dental care have the potential to improve the caries status among ALHIV. In further concordance with our results, poor oral hygiene (including high consumption of sugary drinks and sweets) and poor use to oral health services are well-documented risk factors for dental caries particularly among ALHIV [6, 7, 17].

The OML prevalence in our study is higher than what has been previously reported among similar age cohorts on antiretroviral treatment. A study by da Silva et al., (2008) in Brazil [26] and Moscicki et al. (2016) in the United States of America [6] found the lowest prevalence of OML at <1% while Meless et al. (2014) in West Africa (Mali, Senegal, Ivory Coast) [27] found a prevalence of 8.5%. At least one lesion found in every five ALHIV in our study is not a negligible prevalence and efforts to reduce the OML must continue in our study setting. Frequent screening, early diagnosis and effective management are essential to prevent the progression of symptoms of LGE to a severe form of gingivitis which might result in pain, halitosis and other negative psychosocial and mental consequences such as stigma, shaming or depression [28].

The factors contributing to the presence of OML also varied in many studies. Review papers aimed at providing an update on children and adolescents oral lesions in HIV/AIDS in low and middle income countries, found that the prevalence of OML could vary with diagnostic methods, duration on treatment and treatment regimens [2, 7, 29]. Similarly, we found an association between the presence of OML and the duration on ART, where the longer duration meant less OMLs. Other studies found a correlation of OML with CD4 cell counts <350 cells/mm3 [26, 27] in contrast to the current where only high HIV RNA viral loads more than 1000 copies/ml were significantly associated with OML after adjusting for confounding.

Behavioural traits such as frequent intake of sugary diet and poor oral hygiene habits including infrequent tooth brushing were also found to be directly related to dental caries in the crude analyses but not with OML in the present study. Only good self-rating of oral health was inversely linked to OML. Previous studies have found a correlation of OML with poor oral hygiene [7, 27]. Poor oral hygiene was also related to minimal dental access and poor socio-economic status in the study conducted in Uganda; and has the potential to favour and breed pathogens such as candida spp [27].

The role of ART on OML is a positive one as shown in various literature and in our current study by a lesser prevalence of the lesions [1, 6, 30]. This study also found out that OML such as necrotising ulcerative gingivitis is rare, whilst OMLs such as ulcers (4–16%) and linear gingival erythema (2–9%) are more common in ALHIV. Gaitán-Cepeda *et al*., (2015) found that candidiasis is still prevalent despite ART and it is the highest in African cohorts, followed by Indian cohorts and is lowest in American cohorts (2.9%)[30].

Our study acknowledges the protective effect of HIV treatment and healthy oral hygiene practices on the presence of oral conditions. Consequently, the need to promote treatment adherence and positive oral health behaviour among ALHIV in South Africa is imperative. The distribution of dental caries by socio-demographic characteristics will require further exploration. In all, the correlates of oral conditions identified in this study present at multi-levels and may warrant greater multi-level integration of oral health services into existing adolescents’ HIV treatment, support and care services to better identify and reduce untreated oral conditions.

## Limitations

This study is not without limitations. While this was a cross-sectional survey, it does not indicate if the reported oral conditions are acute or recurrent. Yet, our findings highlight the decreasing but present burden of oral conditions among ALHIV despite their access to HIV treatment, care and support. The reporting of oral health behaviour was self-reported by the participants through an interview with the examiners and may have introduced social desirability and reporting bias. However, the observed associations between the oral health self-report and objective measurements of oral health status gives credibility to the self-reports. Lastly, the reports about the current study should take into cognizance that there was a time lag between the oral examination and or diagnosis of the oral mucosal lesions and dental caries with the HIV blood markers such as HIV RNA viral loads and CD4 counts recorded on the participants’ records of about three weeks.

## Supporting information

S1 Non-identifying data set PLOS ONE YMK *et al*. data set. (XLSX)
